# Characterization of Organic Anion and Cation Transport in Three Human Renal Proximal Tubular Epithelial Models

**DOI:** 10.3390/cells13121008

**Published:** 2024-06-09

**Authors:** Tamara Meijer, Daniel da Costa Pereira, Olivia C. Klatt, Joanne Buitenhuis, Paul Jennings, Anja Wilmes

**Affiliations:** 1 Department of Chemistry and Pharmaceutical Sciences, Vrije Universiteit Amsterdam, De Boelelaan 1108, 1081 HZ Amsterdam, The Netherlands; t.meijer@vu.nl (T.M.); d.dacostapereira@vu.nl (D.d.C.P.); o.c.klatt@vu.nl (O.C.K.); p.jennings@vu.nl (P.J.); 2 Amsterdam Institute of Molecular and Life Sciences (AIMMS), Vrije Universiteit Amsterdam, De Boelelaan 1108, 1081 HZ Amsterdam, The Netherlands

**Keywords:** renal proximal tubule, human induced pluripotent stem cells, organic cation transport, organic anion transport

## Abstract

The polarised expression of specific transporters in proximal tubular epithelial cells is important for the renal clearance of many endogenous and exogenous compounds. Thus, ideally, the in vitro tools utilised for predictions would have a similar expression of apical and basolateral xenobiotic transporters as in vivo. Here, we assessed the functionality of organic cation and anion transporters in proximal tubular-like cells (PTL) differentiated from human induced pluripotent stem cells (iPSC), primary human proximal tubular epithelial cells (PTEC), and telomerase-immortalised human renal proximal tubular epithelial cells (RPTEC/TERT1). Organic cation and anion transport were studied using the fluorescent substrates 4-(4-(dimethylamino)styryl)-N-methylpyridinium iodide (ASP) and 6-carboxyfluorescein (6-CF), respectively. The level and rate of intracellular ASP accumulation in PTL following basolateral application were slightly lower but within a 3-fold range compared to primary PTEC and RPTEC/TERT1 cells. The basolateral uptake of ASP and its subsequent apical efflux could be inhibited by basolateral exposure to quinidine in all models. Of the three models, only PTL showed a modest preferential basolateral-to-apical 6-CF transfer. These results show that organic cation transport could be demonstrated in all three models, but more research is needed to improve and optimise organic anion transporter expression and functionality.

## 1. Introduction

The proximal tubule plays an essential role in the constitutive reabsorption of water and filtered solutes from the glomerular filtrate and the elimination of waste products from the body [1]. To this end, proximal tubular epithelial cells in vivo express a wide range of xenobiotic-handling transporters and metabolising enzymes [2,3]. Due to this, the proximal tubule may be exposed to elevated intracellular levels of potentially harmful substances, rendering it a particularly vulnerable target for toxicity [4,5]. Drug-induced kidney injury can contribute to chronic kidney disease (CKD), which is considered a global health concern, as CKD prevalence continues to rise [6,7,8]. An accurate evaluation of drugs for potential renal toxicity in humans would be greatly improved by the utilisation of a highly predictive model of drug-induced nephrotoxicity that recapitulates human kidney characteristics and closely mimics renal in vivo processes. Traditional approaches to a regulatory safety assessment of chemicals are heavily reliant on animal models, which may poorly predict certain human effects due to species differences in xenobiotic-metabolising and -transport systems and in addition have raised ethical concerns [9,10,11,12]. Therefore, the development of relevant human in vitro renal models that reflect the human renal physiology is advantageous to study and predict drug-induced nephrotoxicity. Notably, human-based in vitro systems can be used as a mechanistic tool to identify cellular and molecular processes underlying drug-induced toxicity [12,13]. Given the significant role of drug transport in the proximal tubule, renal transporters are recognised as major determinants of drug disposition and mediators of drug–drug interactions [1,14]. This highlights the importance of characterising existing and novel human in vitro renal models for their functional transport capacity, with applications for both absorption, distribution, metabolism, and excretion (ADME) and toxicity investigations.

Renal transporters involved in the uptake and efflux of organic cations and anions play a crucial role in drug disposition and the potential development of nephrotoxicity [14]. These transporters can be divided into two major classes, namely the adenosine triphosphate (ATP)-binding cassette (ABC) transporter family, which utilises ATP to drive transport, and the solute carrier (SLC) transporter family, consisting of secondary active transporters dependent on solute (primarily Na^+^) and electrochemical gradients [1,15]. In the renal proximal tubule, the uptake of organic cations is primarily mediated by organic cation transporter 2 (OCT2; *SLC22A2*) expressed at the basolateral membrane [16]. Involved in the apical efflux of organic cations are multidrug and toxin extrusion protein 1 (MATE1; *SLC47A1*) and 2-K (MATE2-K; *SLC47A2*), P-glycoprotein (P-gp; *ABCB1*), and organic cation/carnitine transporter 2 (OCTN2; *SLC22A5*) [2,14,17]. The uptake of organic anions is predominantly mediated by basolaterally expressed organic anion transporter 1 (OAT1; *SLC22A6*) and 3 (OAT3; *SLC22A8*), and the subsequent removal of organic anions into the lumen is mediated by a range of transporters expressed at the apical membrane, including multidrug resistance-associated protein 2 (MRP2; *ABCC2*) and 4 (MRP4; *ABCC4*), breast cancer resistance protein (BCRP; *ABCG2*), and organic anion transporter 4 (OAT4; *SLC22A11*) [2,18,19]. Understanding the interplay between xenobiotics and transporters is pivotal in elucidating the pharmacokinetic and toxicity profiles of drugs, which is why it is desired for a human proximal tubular in vitro model to be equipped with a battery of functional renal transporters involved in drug elimination.

The aim of this study was to investigate the functionality of organic cation and anion transporters in human induced pluripotent stem cell (iPSC)-derived proximal tubular-like cells (PTL), primary human proximal tubular epithelial cells (PTEC), and telomerase-immortalised human renal proximal tubular epithelial cells (RPTEC/TERT1).

## 2. Materials and Methods

### 2.1. Cell Culture

Cryopreserved primary PTEC from two different donors—primary PTEC #1 (batch RPT101031) and primary PTEC #2 (RPT10142)—were purchased from Biopredic International (Parc d’affaires de la Brétèche, Saint-Grégoire, France). The cells were cultured in a 1:1 mixture of Dulbecco’s modified Eagle medium (DMEM) (Gibco, Waltham, MA, USA, 11966-025) and Ham’s F-12 nutrient mix (Gibco, 21765-029) supplemented with 2 mM glutamax (Gibco, 35050-038), 5 µg/mL insulin (Gibco, A11382II), 5 µg/mL transferrin (Sigma-Aldrich, St. Louis, MO, USA, T3705), 5 ng/mL sodium selenite (Sigma-Aldrich, S5261), 10 ng/mL epidermal growth factor (EGF) (Sigma-Aldrich, E9644), 36 ng/mL hydrocortisone (Sigma-Aldrich, H0135), 25 ng/mL prostaglandin E_1_ (Millipore, 538903), 3.4 pg/mL triiodothyronine (Sigma-Aldrich, T5516), and 3.5 µg/mL L-ascorbic acid 2-phosphate (Sigma-Aldrich, A8960). Routinely, cells were cultured in 25 cm^2^ flasks (Thermo Scientific, Waltham, MA, USA) coated with human collagen IV (5 µg/cm^2^) (Sigma-Aldrich, C7521) at 36.5 °C in a 5% CO_2_-humidified environment, fed three times weekly, and subcultured with trypsinisation before confluency was reached. For experiments, cells were plated out into the desired plate formats coated with human collagen IV and cultured for at least 7 days after confluency was reached. Primary PTEC cells were used between passages 1 and 3.

RPTEC/TERT1 cells [20] were obtained from Evercyte GmbH (Vienna, Austria). The cells were cultured in a 1:1 mixture of DMEM and Ham’s F-12 nutrient mix supplemented with 2 mM glutamax, 5 µg/mL insulin, 5 µg/mL transferrin, 5 ng/mL sodium selenite, 10 ng/mL EGF, 36 ng/mL hydrocortisone, 100 U/mL penicillin and 100 µg/mL streptomycin (Sigma-Aldrich, P4333), and 0.5% fetal bovine serum (FBS) (Gibco, 10270-106), at 36.5 °C in a 5% CO_2_-humidified environment, as previously described [21]. For experiments, cells were plated out into the desired plate formats and cultured for a minimum of 10 days after reaching confluency to allow for maturation. In experimental setups, FBS was omitted from the cell culture medium once cells reached confluency. RPTEC/TERT1 cells were used between passages 82 and 92.

The human iPSC lines SBAD2 clone 1 and SBAD3 clone 1 were obtained from the StemBANCC consortium [22]. iPSC were routinely cultured in 6-well plates (Greiner Bio-One) coated with Geltrex^TM^ (8.3 µg/cm^2^) (Gibco, A14133-02) in mTeSR^TM^1 medium (STEMCELL Technologies, 85850) at 36.5 °C in a humidified 5% CO_2_ incubator as previously described [23]. Medium was replaced daily, and iPSC were passaged twice a week using 0.48 mM ethylenediaminetetraacetic acid (EDTA) (Gibco, 15040-066).

The procedures to differentiate iPSC into PTL and to passage differentiated PTL are described in detail in our protocol [23]. The human iPSC lines SBAD2 and SBAD3 were differentiated into PTL within 14 days on Geltrex. On day 14 of the differentiation, PTL were either directly used for subsequent assays or passaged. In case of passaging, cells were plated out into the desired plate formats uncoated or coated with human collagen IV (5 µg/cm^2^) for SBAD2 and SBAD3, respectively. Passaged PTL were used between days 8 and 10 after replating.

Conditionally immortalised human proximal tubular epithelial cell lines ciPTEC, ciPTEC-OAT1, and ciPTEC-OAT3 (Cell4Pharma, Oss, the Netherlands) were cultured as previously described [24,25].

### 2.2. Culturing of Cells on Transwells and Assessment of Barrier Permeability

For barrier permeability and functional transport assays, primary PTEC #1, primary PTEC #2, RPTEC/TERT1, passaged PTL SBAD3, and passaged PTL SBAD2 were cultured on 24-well polyethylene terephthalate (PET) transwells with a pore size of 1 µm (Sarstedt, 83.3932.101) with 100 µL apical and 400 µL basolateral volumes unless specified otherwise, as previously described [23]. When plating out cells, the apical volume was increased from 100 to 200 µL. Apical and basolateral medium were replaced one day after the cells were plated out and then three times weekly.

Integrity of the cell monolayer on transwells was assessed with transepithelial electrical resistance (TEER) measurements and lucifer yellow rejection. TEER was measured using the Epithelial Volt/Ohm Meter EVOM3 (World Precision Instruments). The apical volume was increased to 200 µL to allow for full immersion of the electrodes in cell culture medium. TEER values in Ohms × cm^2^ were obtained by subtracting blank resistance values (transwell without cultured cells) from all samples and multiplying the resulting resistance values by the surface area of the transwell. In addition, transport of lucifer yellow from apical to basolateral side was studied. Apical and basolateral compartments were washed once with Hank’s balanced salt solution (HBSS) (Gibco, 14025-050). Subsequently, cells were incubated with HBSS added to both compartments for 30 min at 37 °C, followed by a 1 h incubation at 37 °C with 60 µM lucifer yellow (Sigma-Aldrich, L0259) in HBSS added to the apical compartment (100 µL) and HBSS only added to the basolateral compartment (200 µL). After 1 h, apical and basolateral supernatant were collected in 96-well plates (Greiner Bio-One). Fluorescence of sample wells as well as of a standard curve was measured at excitation (Ex) 485 nm and emission (Em) 525 nm using the CLARIOstar plate reader (BMG LabTech). Quantification of lucifer yellow with a standard curve was performed in GraphPad Prism using a spline fit. The percentage rejection of lucifer yellow was calculated using the following formula: lucifer yellow % rejection = 100 × (1 − C_BL_/C_AP_), whereby C_BL_ and C_AP_ are the final lucifer yellow concentrations in µM in the basolateral and apical compartments, respectively. Samples were excluded when TEER was lower than 60 Ohms × cm^2^ and when more than 1% lucifer yellow passed through from apical to basolateral side.

### 2.3. Resazurin Reduction Assay

RPTEC/TERT1 and PTL SBAD2 were cultured in 96-well plates. After 24 h exposure to a range of concentrations up to 100 µM of quinidine (Sigma-Aldrich, Q3625), cell viability was evaluated using the resazurin reduction assay as previously described [26]. In brief, supernatant was removed, and cells were incubated with 100 µL/well of 44 µM resazurin in cell culture medium for 1.5 h at 36.5 °C and 5% CO_2_. The reduction of resazurin to resorufin was measured at Ex 540 nm and Em 590 nm using the CLARIOstar plate reader. Relative fluorescence units (RFU) of each well were converted to % control (untreated) after removing the background RFU of the resazurin solution.

### 2.4. Polymerase Chain Reaction (PCR)

Total RNA was isolated from ciPTEC-OAT1, ciPTEC-OAT3, RPTEC/TERT1, PTL SBAD2, and iPSC SBAD2 using the RNeasy Plus Mini Kit (QIAGEN, Hilden, Germany, 74134) according to the manufacturer’s instructions. For primary PTEC #2, the miRNeasy Mini Kit (QIAGEN, 217004) was used. RNA concentration and purity were determined using the NanoDrop 2000 Spectrophotometer (Thermo Scientific). Complementary DNA (cDNA) was synthesised from 1 µg total RNA using iScript Reverse Transcription Supermix (Bio-Rad, Hercules, CA, USA, 1708841) according to the manufacturer’s instructions. PCR reactions were performed using DreamTaq DNA Polymerase (Thermo Scientific, EP0702) according to the manufacturer’s instructions, with 1:100 diluted cDNA and PCR primer concentrations of 0.4 µM final. PCR primers used are shown in Table S1. The recommended thermal cycling conditions were followed with the variable parameters set as follows: the initial denaturation was extended to 10 min, the annealing temperature was set to 55 °C, the number of cycles was 35, and the final extension was set to 10 min. The PCR products were analysed using agarose gel electrophoresis (1.2%) and by staining them with ethidium bromide. The GeneRuler 1 kb Plus DNA Ladder (Thermo Scientific, SM1331) was used to determine the size of the PCR products. Images of the gels were obtained using the Molecular Imager Gel Doc XR+ Imaging System (Bio-Rad).

### 2.5. Immunofluorescence

Primary PTEC #1, RPTEC/TERT1, passaged PTL SBAD3, passaged PTL SBAD2, and iPSC SBAD2 were cultured in PhenoPlate 96-well microplates (PerkinElmer, Waltham, MA, USA). Cell fixation and antibody staining were conducted as previously described [23]. A list of primary and secondary antibodies used is shown in Table S2. Cells were imaged using 40× water (numerical aperture 1.1) or 63× water (numerical aperture 1.15) confocal imaging with the Operetta CLS High-Content Imager (PerkinElmer). Image analysis was performed with the Harmony 4.9 software.

### 2.6. Western Blotting

Cells (primary PTEC #1, RPTEC/TERT1, passaged PTL SBAD3, and iPSC SBAD2) were washed once in cold phosphate-buffered saline (PBS) and then lysed in cold RIPA buffer containing 150 mM NaCl, 5 mM EDTA pH 8.0, 50 mM Tris pH 8.0, 1.0% NP-40, 0.5% sodium deoxycholate, and 0.1% SDS supplemented with 1:100 protease inhibitor cocktail (Sigma-Aldrich, P8340). Samples were incubated for at least 15 min on ice and centrifuged for 10 min at 4 °C at 10,000× *g*, and the insoluble pellets were discarded. Protein content was quantified using the Pierce BCA Protein Assay Kit (Thermo Scientific, 23227) according to the manufacturer’s instructions. Proteins (15 µg) were separated on 12% SDS-PAGE gels and transferred onto methanol-activated 0.2 µm PVDF membranes using the Criterion Blotter (Bio-Rad) under an electrical current of 600 mA for 1.5 h in Towbin buffer (without methanol). Blots underwent three 5 min washes in PBS containing 0.05% Tween (PBS-T), were blocked with 3% bovine serum albumin (BSA) in PBS-T for 30 min at room temperature (RT), and were then incubated overnight at 4 °C with primary antibodies (Table S2) against OCT2 and actin in PBS-T containing 3% BSA. After three 10 min washes in PBS-T, blots were again incubated with 3% BSA in PBS-T for 30 min at RT, followed by a 1 h incubation at RT with corresponding secondary antibodies (Table S2) in PBS-T containing 3% BSA. Following a further three 10 min washes in PBS-T and one wash of 10 min in Tris-buffered saline (TBS), the blots were imaged with the Sapphire Biomolecular Imager (Azure Biosystems) using enhanced chemiluminescence (ECL) substrate (Pierce, Thermo Scientific, 32106).

### 2.7. Functional Transport Assays

To assess functional organic cation transport activity, the substrate 4-(4-(dimethylamino)styryl)-N-methylpyridinium iodide (ASP) (Biotium, 70006) was used alongside the inhibitor quinidine. Primary PTEC #1, primary PTEC #2, RPTEC/TERT1, passaged PTL SBAD3, and passaged PTL SBAD2 were cultured on 24-well PET transwells. Apical and basolateral compartments were washed once with HBSS, and cells were then incubated with HBSS added to both compartments for 30 min at 37 °C. Subsequently, cells were incubated with Hoechst 33342 (1 µg/mL) in HBSS added apically for 20 min at 37 °C. After one wash in HBSS, cells were pre-incubated with or without quinidine (50 µM) in HBSS added basolaterally for 1 h at 37 °C. Next, cells were exposed to ASP (12.5 µM) in HBSS added either basolaterally or apically and with or without quinidine added basolaterally for 1 h at 37 °C. Fluorescence intensity of the substrate ASP was measured both intracellularly and in the apical and basolateral supernatant. The accumulation of ASP in the cells was measured every 10 min until 1 h using 20× air (numerical aperture 0.4) non-confocal imaging with the Operetta CLS High-Content Imager set to 37 °C. Afterwards, apical and basolateral supernatant were collected in 96-well plates, and ASP fluorescence was measured at Ex 485 nm and Em 590 nm using the CLARIOstar plate reader. The images obtained from the Operetta CLS High-Content Imager were analysed with the Harmony 4.9 software to assess ASP accumulation. For each of the samples, the cells were counted utilising the Hoechst 33342 staining. The fluorescence intensity of ASP was calculated only in the areas where Hoechst 33342 staining was detected, after which ASP fluorescence intensity per cell was determined. The blank (HBSS only) was subtracted from all values. The resulting ASP fluorescence intensity per cell values are presented in two formats: either in a time course graph or a one-hour timepoint graph displaying ASP intensity as % control (no inhibitor added). The RFU values of each well containing supernatant collected from the apical compartments were converted to % control after subtraction of the blank RFU.

To assess functional organic anion transport activity, the substrate 6-carboxyfluorescein (6-CF) (Sigma-Aldrich, C0662) and the inhibitor probenecid (Sigma-Aldrich, P8761) were used. ciPTEC-OAT1, ciPTEC, primary PTEC #2, RPTEC/TERT1, and PTL SBAD2 were cultured in 96-well plates. Cells were pre-incubated with or without probenecid (100 µM) in HBSS for 1 h at 37 °C. Next, cells were incubated with 6-CF (1 µM) in HBSS alongside cyclosporine A (CsA) (5 µM) (TCI Chemicals, C2408) and with or without probenecid for 1 h at 37 °C. After two washes in HBSS and subsequent addition of CsA, cells were imaged in the BioTek Cytation 1 Cell Imaging System (Agilent) using a 4× imaging objective and GFP BioTek Filter/LED Cube (Ex 469/35 nm and Em 525/39 nm). The images were obtained with the Gen5 Image Plus Imaging Analysis Software version 3.10.06. Additionally, organic anion transport activity of primary PTEC #1, RPTEC/TERT1, and passaged PTL SBAD3 cultured on 24-well PET transwells was assessed by applying the workflow above as described for organic cation transport using 6-CF and probenecid instead. CsA was not added. 6-CF fluorescence was measured at Ex 485 nm and Em 535 nm.

### 2.8. Statistical Analysis

Data are presented as mean ± standard deviation (SD) for *n* independent experiments given in each figure legend. Statistical analysis was performed using GraphPad Prism. Statistical significance was determined using either one-way ANOVA with Dunnett’s multiple comparisons test or a two-tailed unpaired Student’s *t*-test, as indicated in the figure legends.

## 3. Results

### 3.1. Barrier Integrity

**Figure 1:**
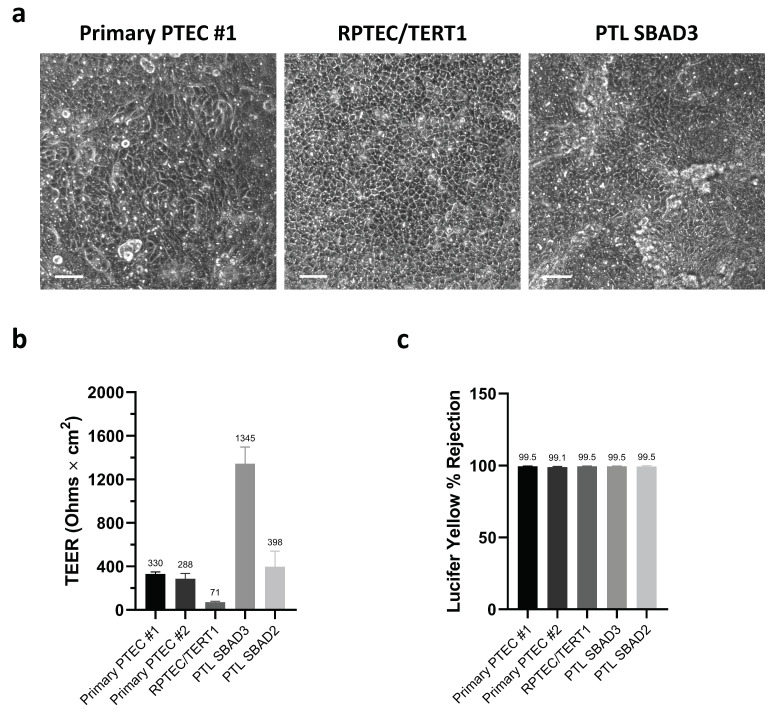
The morphology and tight barrier assessment of primary PTEC, RPTEC/TERT1, and PTL cells cultured on transwells. (**a**) The phase contrast images of matured primary PTEC #1, RPTEC/TERT1, and PTL SBAD3 cultured on transwells. The scale bar represents 50 µm. (**b**) Transepithelial electrical resistance (TEER) measurements of primary PTEC #1, primary PTEC #2, RPTEC/TERT1, PTL SBAD3, and PTL SBAD2 cultured on transwells. (**c**) The barrier integrity assessment by the lucifer yellow rejection of primary PTEC #1, primary PTEC #2, RPTEC/TERT1, PTL SBAD3, and PTL SBAD2 cultured on transwells. Cells were incubated for 1 h with 60 µM lucifer yellow added apically. Both the TEER values and the percentage rejection of lucifer yellow are shown as the mean ± standard deviation (SD) of *n* independent experiments (*n* = 7 for RPTEC/TERT1; *n* = 3 for PTL SBAD3 and SBAD2; *n* = 2 for primary PTEC #1 and #2).

The successful differentiation of iPSC into PTL was qualified by the expression of megalin and the tight junction-associated marker tight junction protein 3 (*TJP3*, also known as ZO-3). PTL cells displayed megalin staining and the expression of ZO-3 exclusively at cell-to-cell borders (Figure S1). Primary PTEC, RPTEC/TERT1, and PTL cells were cultured on transwells, enabling the separation of apical and basolateral compartments. Upon maturation, the cells exhibited a confluent monolayer with a cobblestone-like morphology (Figure 1a). The barrier integrity of the renal models was evaluated by TEER and lucifer yellow rejection. TEER measurements demonstrated the generation of electrical resistance across all cell monolayers (Figure 1b). Additionally, less than 1% lucifer yellow passed through from the apical to the basolateral side (Figure 1c), indicating the formation of tight monolayers on transwells.

Furthermore, we tested whether the inhibitor quinidine could compromise the barrier integrity by inducing cellular toxicity. Quinidine did not significantly affect resazurin reduction in RPTEC/TERT1 and PTL cells up to 50 and 100 µM, respectively, after 24 h exposure (Figure S2).

### 3.2. Expression of Organic Cation Transporters

**Figure 2:**
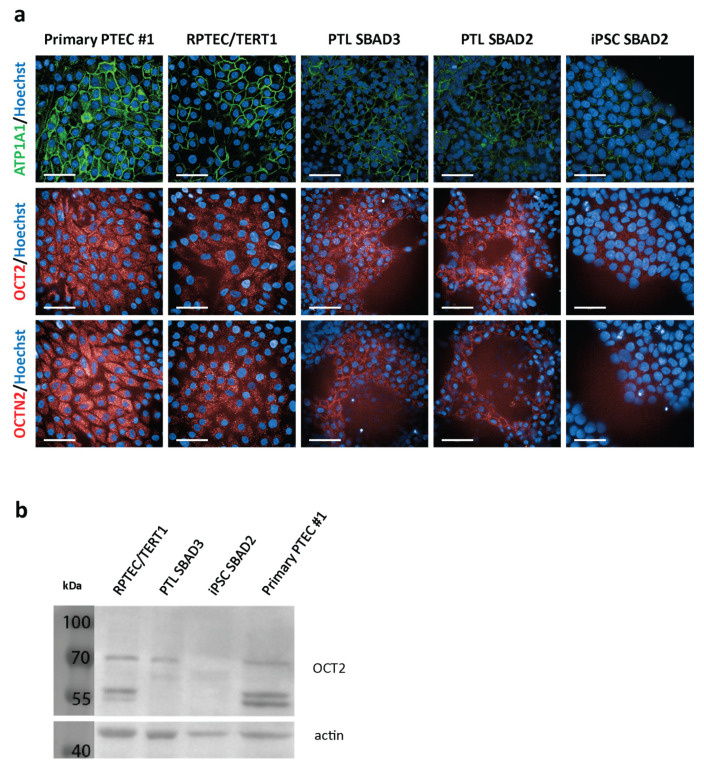
The expression of proteins involved in organic cation transport. (**a**) The immunofluorescent images of primary PTEC #1, RPTEC/TERT1, PTL SBAD3, PTL SBAD2, and iPSC SBAD2 stained with the nuclear stain Hoechst 33342 (blue) and antibodies against ATPase Na^+^/K^+^ transporting subunit alpha 1 (ATP1A1) (green), organic cation transporter 2 (OCT2) (red), and organic cation/carnitine transporter 2 (OCTN2) (red). Images were obtained using 63× water confocal imaging. Auto contrast was set per antibody staining to allow for a comparison between the different cell models. The scale bar represents 50 µm. (**b**) The Western blot of OCT2 in primary PTEC #1, RPTEC/TERT1, PTL SBAD3, and iPSC SBAD2. Actin was used as an internal control.

The protein expression of OCT2 and OCTN2 in primary PTEC, RPTEC/TERT1, and PTL cells was analysed with immunofluorescence and/or Western blotting. Undifferentiated iPSC were used as a negative control. Antibody staining could detect OCT2 and OCTN2 in all renal cell systems and was at comparable levels between primary PTEC, RPTEC/TERT1, and PTL cells (Figure 2a). In addition, primary PTEC, RPTEC/TERT1, and PTL showed a strong expression of ATPase Na^+^/K^+^ transporting subunit alpha 1 (ATP1A1), whereas undifferentiated iPSC expressed low levels of ATP1A1 (Figure 2a). The Western blot analysis revealed a double band at approximately 55 and 60 kDa and a higher band at approximately 70 kDa for OCT2 in primary PTEC and RPTEC/TERT1 cells (Figure 2b), which is similar to previously reported results in RPTEC/TERT1 cells, of which the higher band is suggested to be the result of *N*-glycosylation [27,28]. In PTL, only this high band was observed, and no OCT2 staining could be detected in undifferentiated iPSC (Figure 2b).

### 3.3. Organic Cation Transport Activity

**Figure 3:**
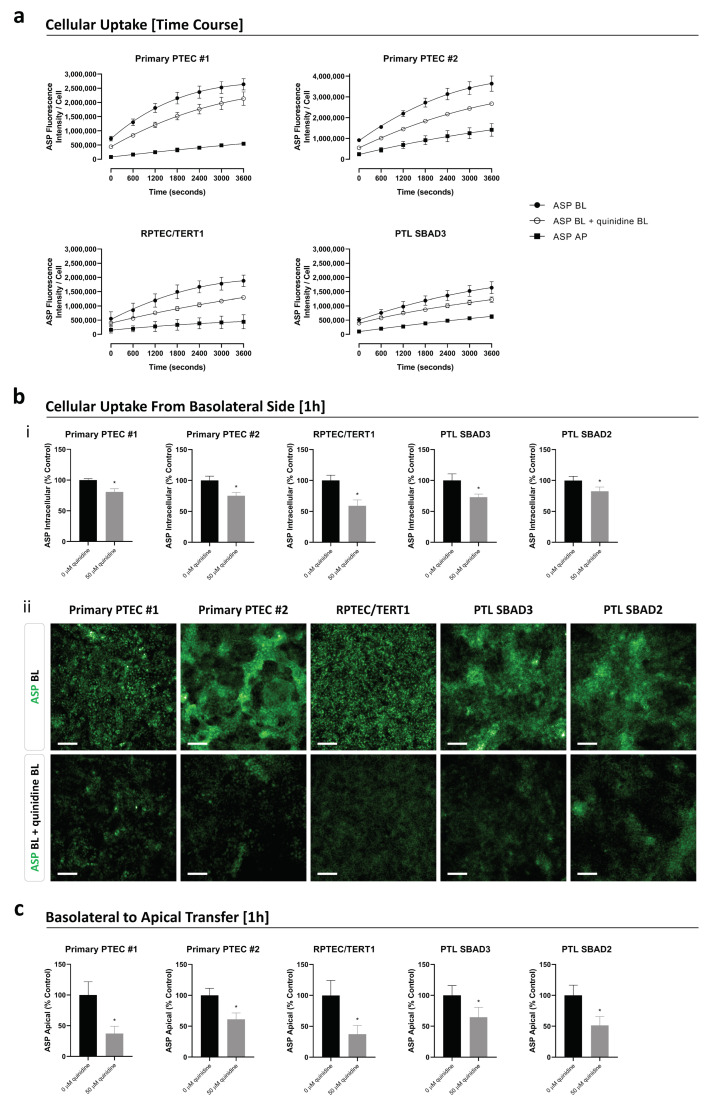
The uptake and transfer of the fluorescent substrate 4-(4-(dimethylamino)styryl)-N-methylpyridinium iodide (ASP) in primary PTEC, RPTEC/TERT1, and PTL cells cultured on transwells. Cells were pre-incubated for 1 h in the absence or presence of the inhibitor quinidine applied basolaterally (BL), followed by a 1 h incubation with the substrate ASP applied BL or apically (AP) in the absence or presence of quinidine applied BL. (**a**) The time-dependent accumulation of ASP in cells in the absence or presence of quinidine shown as ASP fluorescence intensity per cell. Data are shown as the mean ± SD of 2 independent experiments. A two-tailed unpaired Student’s *t*-test was performed between the area under the curves (AUCs) of the three different conditions, which were all significantly different from one another in all cell models (*p*-value < 0.05). (**b**-**i**) The accumulation of ASP in cells in the absence or presence of quinidine, all applied BL, after 1 h incubation. Data are shown as the mean ± SD of *n* independent experiments (*n* = 4 for RPTEC/TERT1; *n* = 3 for PTL SBAD3 and SBAD2; *n* = 2 for primary PTEC #1 and #2), depicted as % control (no inhibitor added). Statistical significance was calculated using a two-tailed unpaired Student’s *t*-test. * represents a *p*-value < 0.05 (no inhibitor versus inhibitor added). (**b-ii**) The representative images of ASP accumulation in cells after 1 h incubation with or without quinidine. To visualise the best effect of the inhibitor, auto contrast was set per cell type to compare ASP accumulation with and without an inhibitor. The scale bar represents 100 µm. (**c**) The transport of ASP from basolateral to apical supernatant in the absence or presence of quinidine applied BL after 1 h incubation. Data are shown as the mean ± SD of *n* independent experiments (*n* = 4 for RPTEC/TERT1; *n* = 3 for PTL SBAD3 and SBAD2; *n* = 2 for primary PTEC #1 and #2), depicted as % control (no inhibitor added). Statistical significance was determined as described under (**b**-**i**).

The functionality of renal transporters involved in organic cation transport was evaluated in cells cultured on transwells by measuring the uptake or transfer of the fluorescent substrate ASP in the absence and presence of the inhibitor quinidine. The application of the substrate ASP to either the apical or basolateral side resulted in a time-dependent increase in intracellular ASP levels in primary PTEC, RPTEC/TERT1, and PTL cells (Figure 3a). All models exhibited significantly higher intracellular levels of ASP when applied basolaterally rather than apically (Figure 3a), demonstrating that ASP uptake was favoured from the basolateral rather than the apical side. After 1 h of basolateral application, the amount of ASP in primary PTEC cells from donor one and donor two was approximately 1.4-fold and 1.9-fold higher, respectively, compared to RPTEC/TERT1 cells and approximately 1.6-fold and 2.2-fold higher compared to PTL cells (Figure 3a). Within the initial 10 min of basolateral application, the intracellular levels of ASP increased approximately 1.9-fold and 2.1-fold faster in primary PTEC cells from donors one and two, respectively, compared to RPTEC/TERT1 cells and approximately 2.3-fold and 2.6-fold faster compared to PTL cells (Figure 3a). The basolateral uptake of ASP could be inhibited by the basolateral application of the inhibitor quinidine in all models (Figure 3a,b). After a 1 h co-incubation of the substrate and inhibitor, the intracellular ASP levels were reduced to 59 ± 9% in RPTEC/TERT1, 73 ± 5% in PTL SBAD3, 75 ± 5% in primary PTEC from donor two, 81 ± 5% in primary PTEC from donor one, and 82 ± 7% in PTL SBAD2 (Figure 3b). In addition to measuring intracellular ASP levels, the amount of ASP in the apical supernatant was assessed to account for efflux via organic cation transporters located on the apical membrane. All tested proximal tubular cell models demonstrated uptake of ASP from the basolateral side with its subsequent release into the apical compartment. This process could be inhibited by basolateral exposure to quinidine, whereby the ASP levels in the apical supernatant were reduced to 37 ± 12% for primary PTEC from donor one, 37 ± 14% for RPTEC/TERT1, 51 ± 14% for PTL SBAD2, 61 ± 10% for primary PTEC from donor two, and 65 ± 16% for PTL SBAD3 (Figure 3c). The amount of ASP in the basolateral supernatant after apical application was also measured and compared to basolateral-to-apical ASP transfer. Although some ASP was transported from the apical to basolateral side, the amount was substantially less, demonstrating that the preferred direction of ASP transfer was clearly basolateral to apical (Figure S3).

### 3.4. Expression and Functionality of Organic Anion Transporters

**Figure 4:**
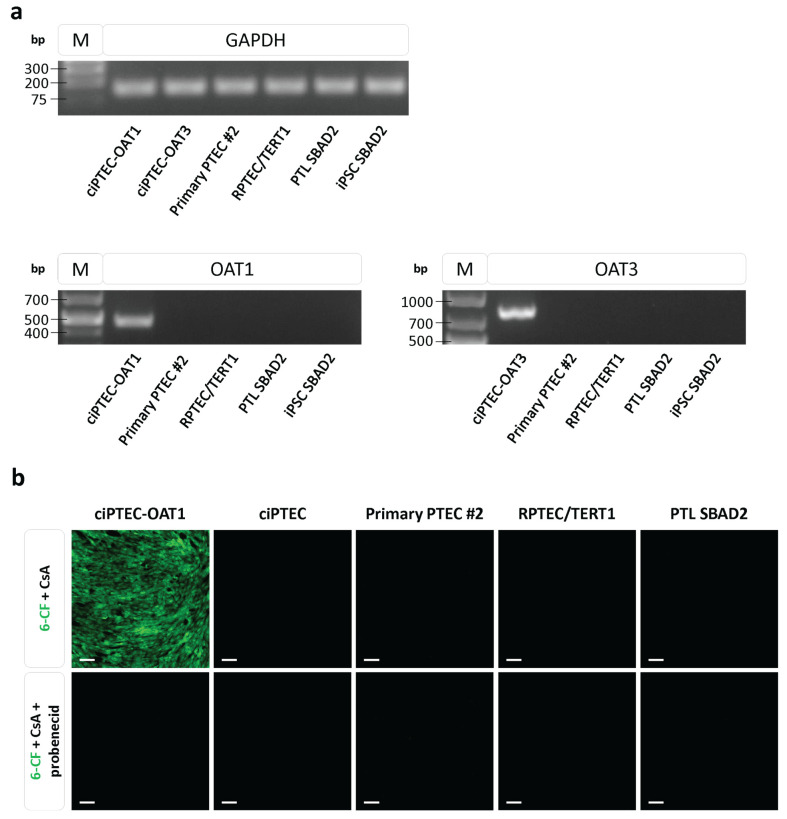
The expression and function of organic anion transporter 1 (OAT1) and 3 (OAT3). (**a**) The mRNA expression of OAT1 and OAT3 in ciPTEC-OAT1, ciPTEC-OAT3, primary PTEC #2, RPTEC/TERT1, PTL SBAD2, and iPSC SBAD2. Glyceraldehyde-3-phosphate dehydrogenase (*GAPDH*) served as an internal control. Representative images from the gel electrophoresis of polymerase chain reaction (PCR) products are shown. (**b**) The cellular uptake of the fluorescent substrate 6-carboxyfluorescein (6-CF). ciPTEC-OAT1, ciPTEC, primary PTEC #2, RPTEC/TERT1, and PTL SBAD2 were cultured on microplates. Cells were pre-incubated for 1 h in the absence or presence of the inhibitor probenecid, followed by a 1 h incubation with the substrate 6-CF alongside cyclosporine A (CsA) to block efflux and in the absence or presence of probenecid. The uptake of 6-CF was determined using the BioTek Cytation 1 Cell Imaging System with a 4× imaging objective. Representative images are shown. The scale bar represents 100 µm.

The expression and functionality of organic anion transporters in the renal models were investigated using the OAT-transduced cell lines ciPTEC-OAT1 and ciPTEC-OAT3 as positive controls. ciPTEC-OAT1 and ciPTEC-OAT3 exhibited OAT1 and OAT3 mRNA expression, respectively, whereas no OAT1 and OAT3 mRNA could be detected in primary PTEC, RPTEC/TERT1, PTL, and undifferentiated iPSC (Figure 4a).

For the assessment of functional organic anion transport, the uptake of the fluorescent substrate 6-CF in the absence and presence of the inhibitor probenecid was studied in all cell systems, using the ciPTEC-OAT1 cells as a positive control. The OAT transport assay was performed with cells cultured on plastic, as previously reported [25]. To evaluate the accumulation of substrate within the cells, CsA was added to reduce efflux via ABC transporters [14,29]. When cultured on plastic, only ciPTEC-OAT1 demonstrated cellular uptake of the substrate 6-CF, which could be subsequently inhibited by probenecid, whereas no uptake could be detected in ciPTEC, primary PTEC, RPTEC/TERT1, and PTL cells (Figure 4b). The basolateral side is not easily accessible in fully polarised epithelial cells that are cultured on plastic; therefore, primary PTEC, RPTEC/TERT1, and PTL were also cultured on transwells to study basolateral-to-apical and apical-to-basolateral transfer without the addition of CsA. In only iPSC-derived PTL, the transfer of 6-CF, albeit small, seemed to be favoured from the basolateral to apical side; however, this transfer was not significantly inhibitable by probenecid (Figure S4).

## 4. Discussion

Various different human in vitro renal proximal tubular models have been successfully employed for nephrotoxicity studies, including immortalised cell lines (e.g. HK-2, RPTEC/TERT1, and ciPTEC), primary cells (i.e. cells cultured directly from tissue and not immortalised), and, more recently, iPSC-derived cells (either as multicellular organoids or monocultures targeting a specific cell type) [24,25,30,31,32,33]. Each of these models has certain advantages and disadvantages, which include non-trivial issues, such as differentiation status, accessibility (including licensing and costs of acquisition), ease of use, and/or the ability to deliberately select donors. It is a commonly held assumption that primary cells, when isolated from healthy material and cultured under optimal conditions, would best represent the differentiation status of proximal tubule cells. This assumption is based on the fact that cells are already differentiated at the time of isolation but is complicated by the method of isolation, culture conditions, medium, and the use of antimicrobial agents. Cell lines, which usually have more standardised culture conditions and are better characterised, have already dedifferentiated to a certain level and have issues with regard to immortalisation, which by definition affects the operation of critical pathways, such as p53, and can prevent or disturb contact inhibition, an important part of epithelial and proximal tubule function. However, more selective immortalisation techniques, for example, via the overexpression of the catalytic region of telomerase as used in the RPTEC/TERT1 cell line, allow for immortalisation without a negative impact on contact inhibition [20,32]. These cells become contact-inhibited to form a functional transporting monolayer, which is characterised by dome formation on non-porous growth supports, and present a consistent solute and water transporting barrier when cultured on microporous growth supports, a.k.a. transwells [32,34]. Nevertheless, a drawback of using cell lines for toxicity studies is that genetic variability is not accounted for, as traditional cell lines represent one genetic background only. The ability to easily generate iPSC from somatic nucleated cells uniquely allows for differentiation to target cells from any given donor [35,36] and as such are hugely attractive to pharmacological and toxicological studies. There are several different strategies for iPSC differentiation into renal lineages that use specific cell culture conditions, small molecules, and growth factors to simulate developmental processes, including transient manipulation of retinoic acid and WNT signalling pathways as well as growth factor-specific processes. In the present study, we have utilised a previously characterised two-week strategy to differentiate iPSC cells into proximal tubular-like cells (PTL) [23,33]. These cells exhibit certain characteristics of proximal tubule cells, such as the megalin-mediated uptake of albumin and functional P-gp activity [33], and have been shown to be a promising model for toxicity prediction and assessment in several transcriptomic studies [37,38,39,40].

Since organic cation and organic anion uptake processes contribute to the internal proximal tubule exposure to many dietary and pharmaceutical compounds, the ability of the cell culture model to perform these tasks is considered important. Thus, we endeavoured to characterise the activity of these processes using three different types of human proximal tubule models, namely primary, cell line, and iPSC-derived. We utilised the organic cation fluorescent compound ASP and the organic anion fluorescent compound 6-CF to measure both the uptake and basolateral-to-apical (or vice versa) transfer of these fluorescent compounds in cells cultured as monolayers on transwells. These assays were conducted after the differentiation of the cells on the transwells, measuring cellular uptake and quantifying the fluorescent compounds in the apical and basolateral medium over a period of an hour. The experiments were conducted with the apical compartment positioned on top and the basolateral compartment on the bottom. Additionally, quinidine (50 µM) and probenecid (100 µM) were used to inhibit organic cation and organic anion transport, respectively.

All three models demonstrated quinidine-inhibitable ASP uptake from the basolateral compartment and basolateral-to-apical transport. Organic cation transport activity in PTL was lower but still within a 3-fold range compared to primary PTEC and RPTEC/TERT1 cells. In the opposite direction, the cellular uptake and transfer of ASP were substantially less in all renal models. Such results would only be expected if the preferential directionality of the transfer is from the basolateral to the apical side (i.e., from interstitium to lumen). It is expected that basolateral organic cation uptake is mediated by OCT2, which is predominantly expressed in the proximal tubule [41]. Immunofluorescence and Western blot staining provide evidence for OCT2 expression in all cell models.

Organic anion transport activity was not measurable via the cellular uptake of 6-CF in the three cell types investigated when cultured on plastic. Although due to the proven polarity of these models, such a result could be expected where basolateral uptake is predominant. On transwells, PTL was the only model that displayed modest transfer of 6-CF that seemed to be favoured from the basolateral to apical side but could not be significantly inhibited. Future work is needed to further improve this activity in the PTL model. A low expression or the absence of the functional expression of organic anion transporters is a commonly reported issue in renal in vitro cell models [42,43,44,45]. Caetano-Pinto et al. [42] demonstrated a lack of OAT1 and OAT3 mRNA in primary proximal tubular cells cultured under static conditions, while OCT2 expression was comparable to that of the kidney cortex. Other studies by Zhang et al. [43] and van der Hauwaert et al. [44] also showed a lack of OAT1 and OAT3 mRNA in primary cell cultures, whereas the expression of OATs was observed in human kidney tissue. Nonetheless, there are studies that did report the functional expression of organic anion transporters in primary cells that were cultured on transwells [46,47,48]. The reasons for the variability in the expression of OATs among primary isolates may be related to several factors, such as isolation techniques, the duration of isolation, cell culture methods, population doublings until time of use, the passage number, etc. Since these were not systematically investigated, it is difficult to pinpoint the exact reasons. Variabilities among donors may also contribute to variations in OAT expression and function. This was exemplified by Lash et al. [46], who showed significant variability in the protein expression of OAT1 and OAT3 across human kidney plasma membranes from eight different donors. Another study by Chapron et al. [49] demonstrated differences in OAT function between primary cells from two different donors, whereby one donor showed much lower transport of the substrate *p*-aminohippuric acid (PAH) compared to the other donor.

The consistent expression of OATs appears to be challenging even in primary PTEC cells, which highlights the need for the optimisation of culture methodologies to maintain OAT expression and functionality in renal cultures. Unfortunately, the mechanisms behind the loss of OAT1 and OAT3 expression in vitro remain largely unknown. This could be due to inappropriate medium composition, the presence of suppressors, and/or the lack of inducers. Alternatively, the nutrient conditions and oxygen saturation in the medium might be sub-optimal, or the culture of cells on plastic supports (which is standard for the first primary isolates) may permanently deregulate some genes. Since hepatocyte nuclear factors HNF1A and HNF4A are thought to play an important role in OAT regulation [50,51], manipulating this network might aid in the re-expression of these transporters. The utilisation of microphysiological systems (MPS) could also offer a solution since these systems may provide more physiological culture conditions with respect to flow sensing, nutrient delivery, and oxygen saturation. Indeed, some studies have shown that OATs are expressed when primary cells are cultured under microfluidic conditions [49,52]. For instance, primary PTEC have been demonstrated to exhibit probenecid-inhibitable PAH transepithelial transport in an MPS device but not when maintained under static conditions [52]. However, other studies have shown that MPS systems did not improve OAT functionality [42,53]. Nevertheless, MPS systems could offer a promising avenue for further exploration for such studies, including improving the differentiated phenotype of iPSC-derived PTL.

With regard to iPSC-derived renal models, aside from the iPSC-derived monocultures of proximal tubular cells that we described in the present study, several protocols have been reported that describe the differentiation of human iPSC into kidney organoids [54,55,56,57,58,59,60,61,62,63,64] containing multiple cell phenotypes, with application for developmental studies and disease modelling. Renal organoids are a promising in vitro model as we could enhance our understanding of renal physiology by studying the interactions between the different cell phenotypes within an organoid. In this respect, they might be more physiologically relevant compared to monocultures, maybe also concerning the expression of OATs. However, they also have limitations, including high numbers of off-target cells, such as neurons and muscle cells, comprising approximately 11 to 22% of the kidney organoid [59,62,65]. A study by Lawrence et al. [59] reported the functionality of organic cation and anion transporters in 3D organoids using specific substrates and inhibitors. Furthermore, Shankar et al. [60] demonstrated functional organic anion transport in renal organoids at 37 °C using the substrate fluorescein, which was inhibited at 4 °C. In other studies, the expression levels of OCT2, OAT1, and OAT3 were shown; however, these transporters were not characterised functionally [56,63,64]. It is more challenging to comprehensively characterise functional transport activity in these multicellular 3D cultures, as there is no easy access to the apical side.

## 5. Conclusions

In conclusion, we could show that iPSC-derived PTL, human primary PTEC, and RPTEC/TERT1 cells exhibited functional organic cation transport, thus making these models suitable for nephrotoxicity and transport studies involving compounds handled by organic cation transporters. More work is required to investigate OAT regulation mechanisms in order to potentially restore this function in primary cells and cell lines and to further induce it in iPSC-derived proximal tubule cells.

## Data Availability

Data are available on reasonable request from the authors.

## Supplementary Materials

The following supporting information can be downloaded at https://www.mdpi.com/article/10.3390/cells13121008/s1, Table S1: List of PCR primers; Table S2: List of primary and secondary antibodies used for immunofluorescence (IF) and Western blotting (WB); Figure S1: The immunofluorescent images of the proximal tubular-enriched marker megalin and tight junction marker tight junction protein 3 (*TJP3*, aka ZO-3) in PTL; Figure S2: Effect of quinidine on cell viability; Figure S3: ASP basolateral-to-apical versus apical-to-basolateral transfer; Figure S4: 6-CF basolateral-to-apical versus apical-to-basolateral transfer.
