# Supplementary material for: Characterization of Organic Anion and Cation Transport in Three Human Renal Proximal Tubular Epithelial Models
Source: Cells. 2024 Jun 9;13(12):1008. doi: 10.3390/cells13121008 (PMC11202273; doi:10.3390/cells13121008)
Supplement: Supplementary file 1 [file cells-13-01008-s001.zip › cells-3050517-supplementary.pdf]

Supplementary Materials

Table S1: List of PCR primers. Sequences of forward and reverse primers used for PCR with resulting PCR product size. The housekeeping gene *GAPDH* served as internal control.

Gene	Sequence Forward Primer	Sequence Reverse Primer	Product Size (bp)
**GAPDH**	5'- GTC TCC TCT GAC TTC AAC AGC G -3'	5'- ACC ACC CTG TTG CTG TAG CCA A -3'	131
**SLC22A6 (OAT1)**	5'- GGC TTC CTT GTC ATC AAC TCC C -3'	5'- ATA CTT CTG GTG CTC TTG TTG CTG -3'	456
**SLC22A8 (OAT3)**	5'- CAA CAG CAC CAA GGA CTC CAT TG -3'	5'- GAG GTT GAC TCC AAA TTC TTC CAC ACC -3'	754

Table S2: List of primary and secondary antibodies used for immunofluorescence (IF) and western blotting (WB).

Name	Supplier	Catalogue Number	Dilution	Application
**Actin**	Santa Cruz Biotechnology	sc-8432	1:1000	WB
**ATP1A1**	Invitrogen	MA3-928	1:100	IF
**Donkey anti-mouse Alexa Fluor 488**	Invitrogen	A21202	1:1000	IF
**Donkey anti-rabbit Alexa Fluor 546**	Invitrogen	A10040	1:1000	IF
**Donkey anti-rabbit Alexa Fluor Plus 647**	Invitrogen	A32795	1:1000	IF
**Goat anti-mouse HRP**	Abcam	ab6789	1:5000	WB
**Goat anti-rabbit HRP**	Abcam	ab6721	1:5000	WB
**Hoechst 33342 (nuclear stain)**	Invitrogen	H3570	1:10000 (final: 1 µg/mL)	IF
**Megalin**	R&D systems	MAB9578	1:100	IF
**OCT2**	Sigma-Aldrich	HPA008567	1:200	IF
**OCT2**	Sigma-Aldrich	HPA008567	1:500	WB
**OCTN2**	Abcam	ab79964	1:100	IF
**ZO-3**	Cell Signaling Technology	3704	1:1600	IF

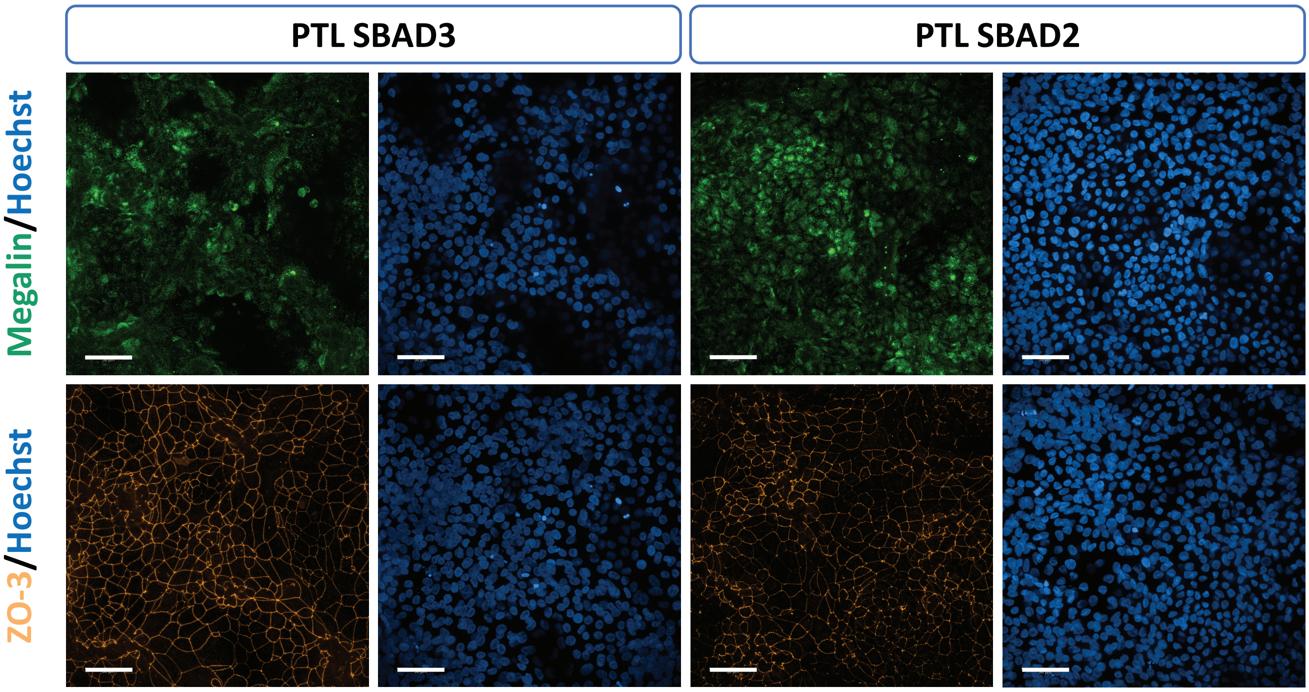

Figure S1: Immunofluorescent images of the proximal tubular-enriched marker megalin and tight junction marker tight junction protein 3 (*TJP3*, aka ZO-3) in PTL. PTL SBAD3 and SBAD2 were stained with the nuclear stain Hoechst 33342 (blue) and antibodies against megalin (green) and ZO-3 (orange). Images were obtained using 40× water confocal imaging. The scale bar represents 50 µm.

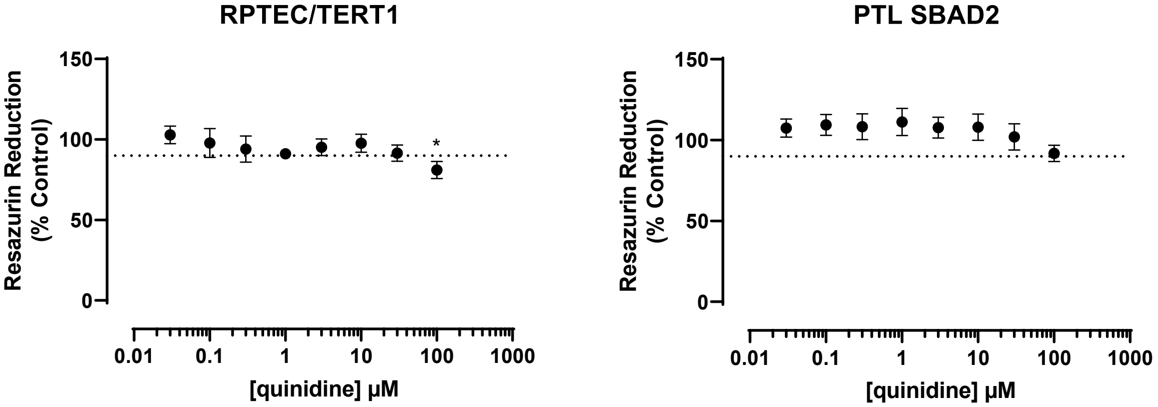

Figure S2: Effect of quinidine on cell viability. RPTEC/TERT1 and PTL SBAD2 cells were exposed to quinidine for 24 h. Cell viability was assessed after 1.5 h incubation in 44 µM resazurin. Data is shown as mean ± SD of 2 independent experiments, depicted as % control (untreated). Statistical significance was calculated using one-way ANOVA. * represents *p*-value < 0.05 (untreated versus treated) and ≥ IC_{10}. The dotted line represents a decrease of 10% in resazurin reduction compared to control.

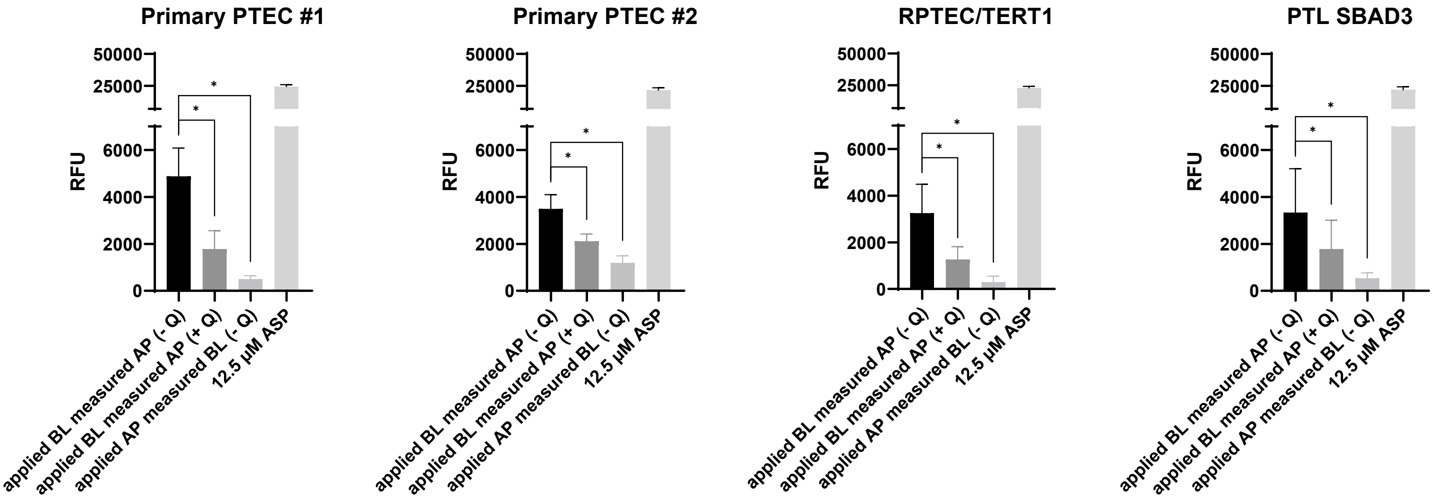

Figure S3: ASP basolateral to apical versus apical to basolateral transfer. Cells were pre-incubated for 1 h in absence or presence of the inhibitor quinidine (Q) applied BL, followed by a 1 h incubation with the substrate ASP applied BL or AP in absence or presence of the inhibitor applied BL. Relative fluorescence units (RFU) of the applied 12.5 µM ASP is shown as comparison. RFU data is shown as mean ± SD of 2 independent experiments minimum. Statistical significance was calculated using a two-tailed unpaired Student's *t*-test. * represents p-value < 0.05.

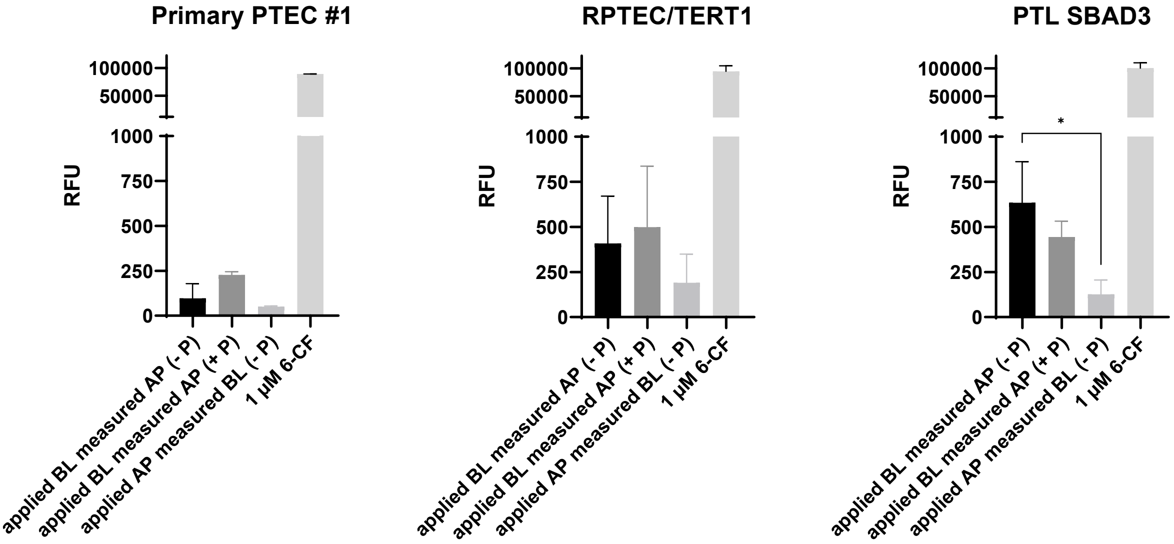

Figure S4: 6-CF basolateral to apical versus apical to basolateral transfer. Cells were pre-incubated for 1 h in absence or presence of the inhibitor probenecid (P) applied BL, followed by a 1 h incubation with the substrate 6-CF applied BL or AP in absence or presence of the inhibitor applied BL. RFU of the applied 1 µM 6-CF is shown as comparison. RFU data is shown as mean ± SD of 2 independent experiments minimum. Statistical significance was calculated using a two-tailed unpaired Student's *t*-test. * represents *p*-value < 0.05.
